# Supplementary figures and images for: Daidzein alleviates osteoporosis by promoting osteogenesis and angiogenesis coupling
Source: PeerJ. 2023 Oct 16;11:e16121. doi: 10.7717/peerj.16121 (PMC10586307; doi:10.7717/peerj.16121)

Figure3G

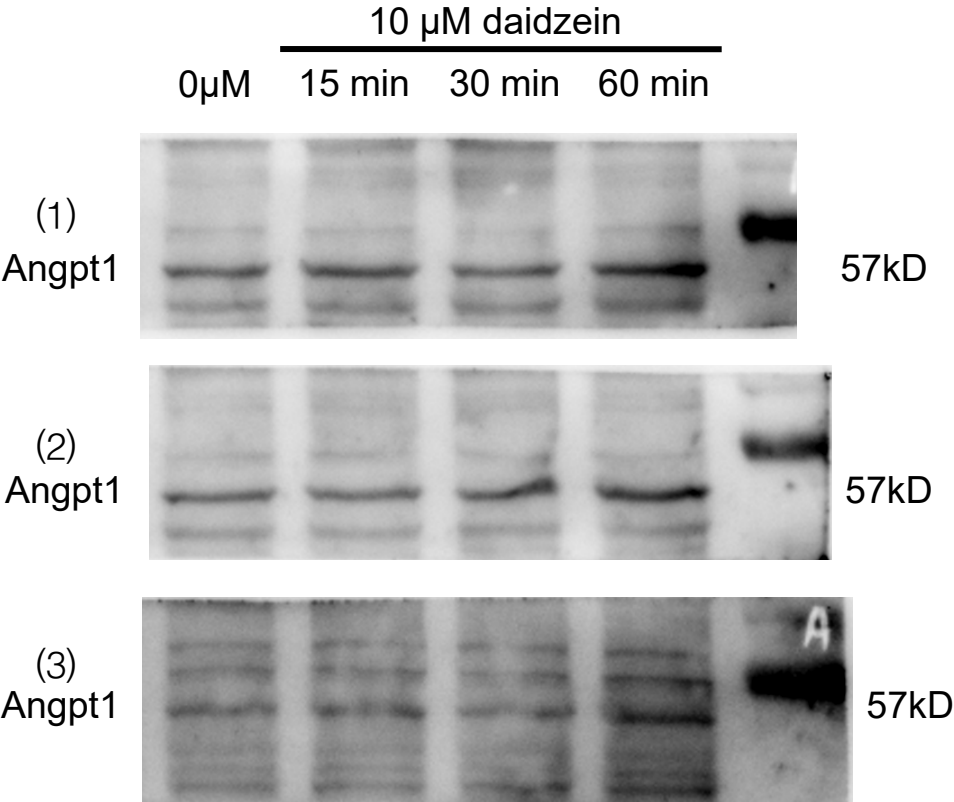

Figure3G

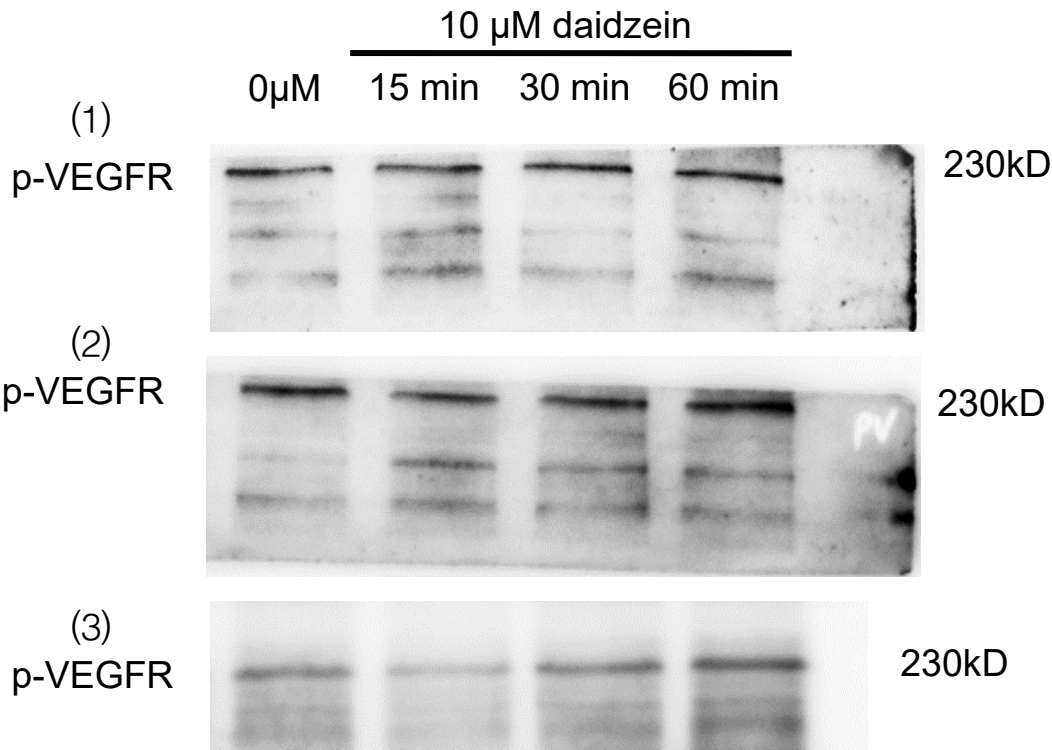

Figure3G

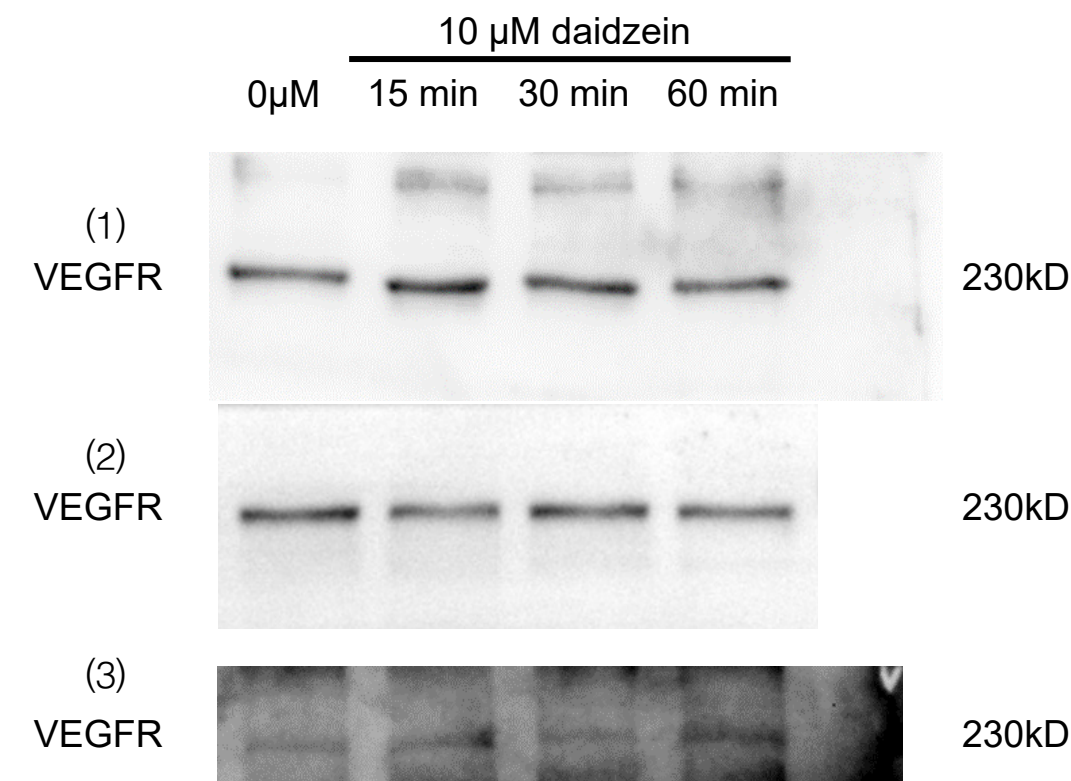

Figure3G

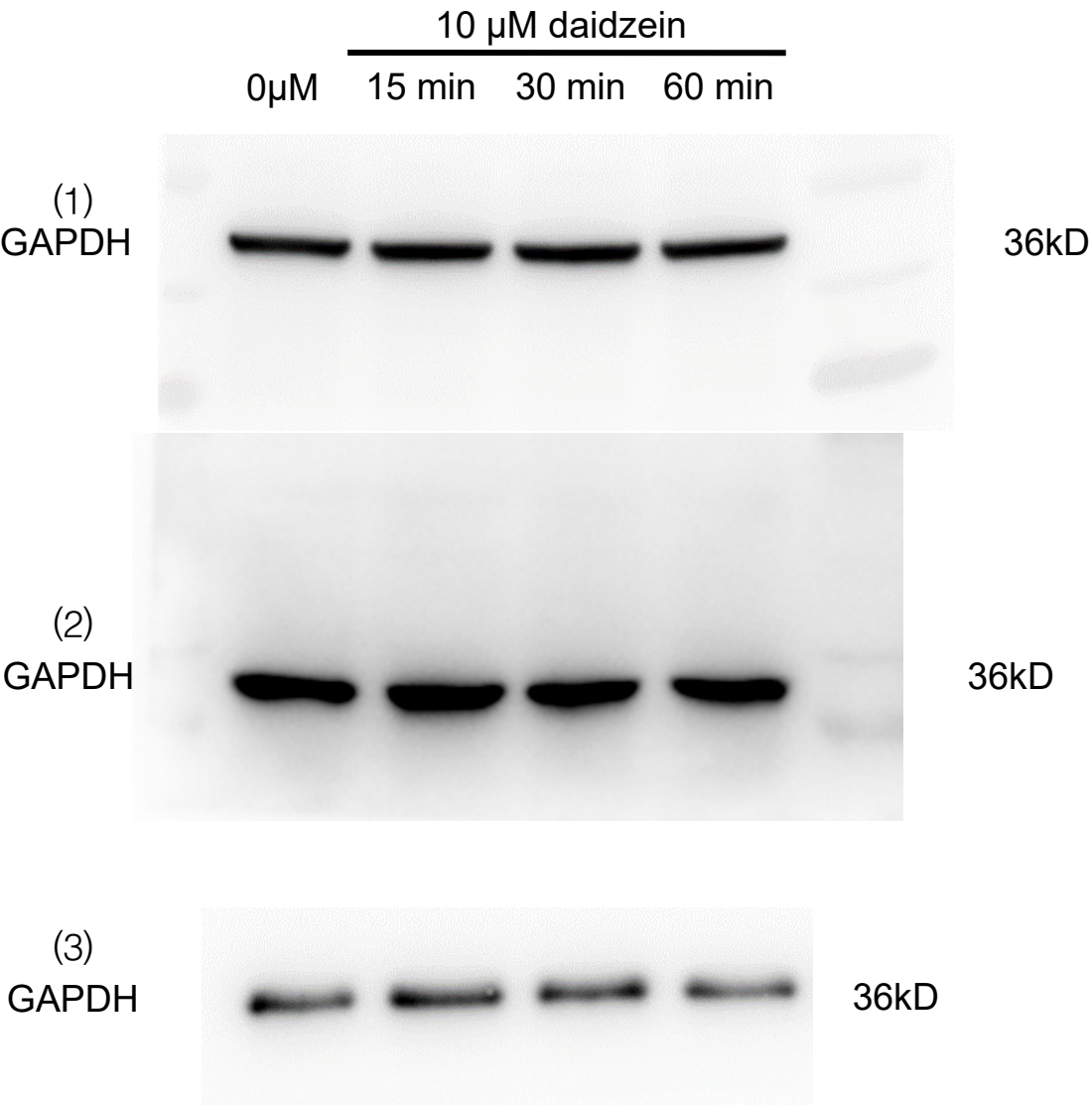

Supplement: Supplemental Information 3 [file peerj-11-16121-s003.pdf]
