# Supplementary material for: Daidzein alleviates osteoporosis by promoting osteogenesis and angiogenesis coupling
Source: PeerJ. 2023 Oct 16;11:e16121. doi: 10.7717/peerj.16121 (PMC10586307; doi:10.7717/peerj.16121)
Supplement: Supplemental Information 4 [file peerj-11-16121-s004.pdf]

Figure 4A

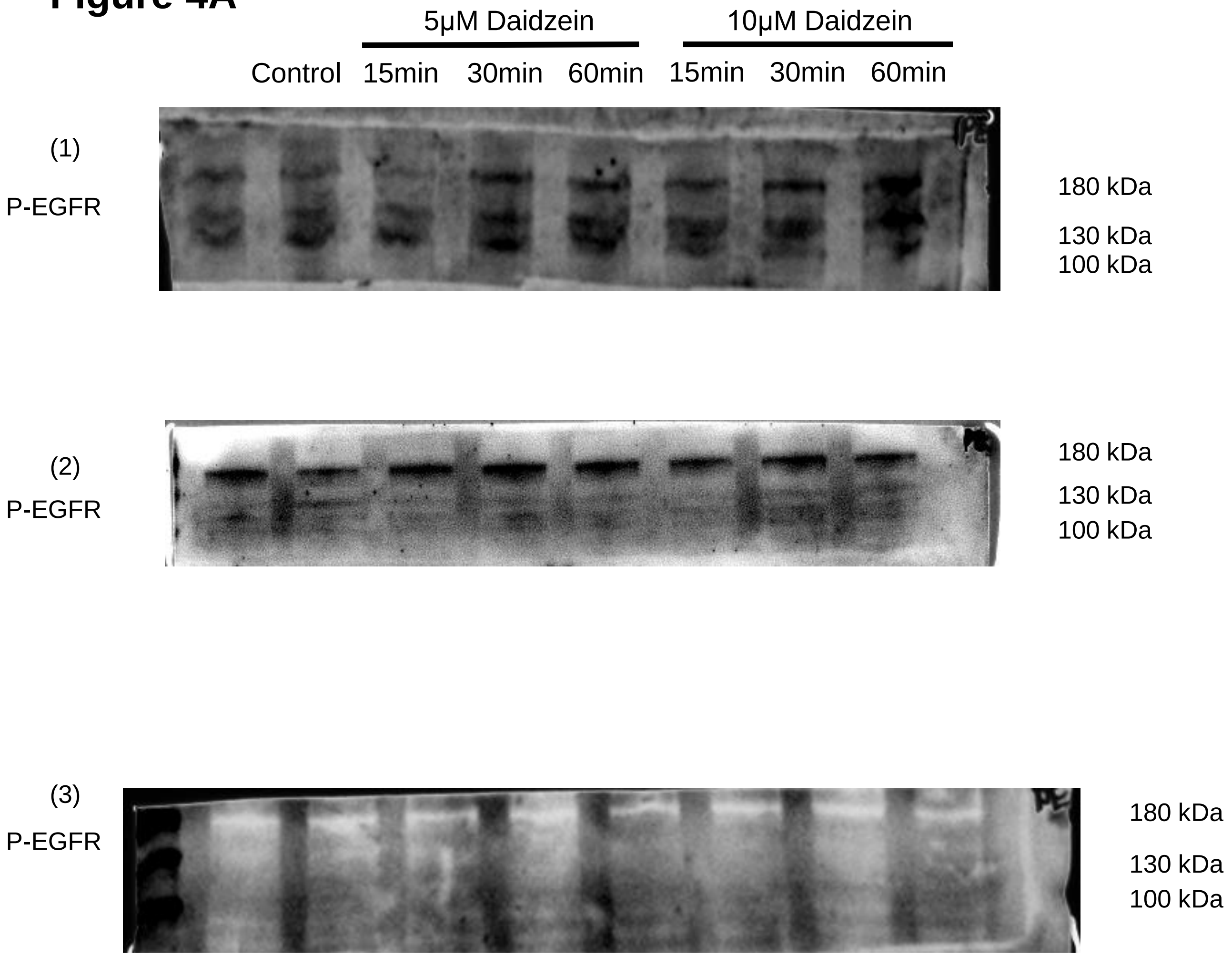
5μM Daidzein
10μM Daidzein
Control
15min
30min
60min
15min
30min
60min
(1)
P-EGFR
180 kDa
130 kDa
100 kDa
(2)
P-EGFR
180 kDa
130 kDa
100 kDa
(3)
P-EGFR
180 kDa
130 kDa
100 kDa

Figure 4A

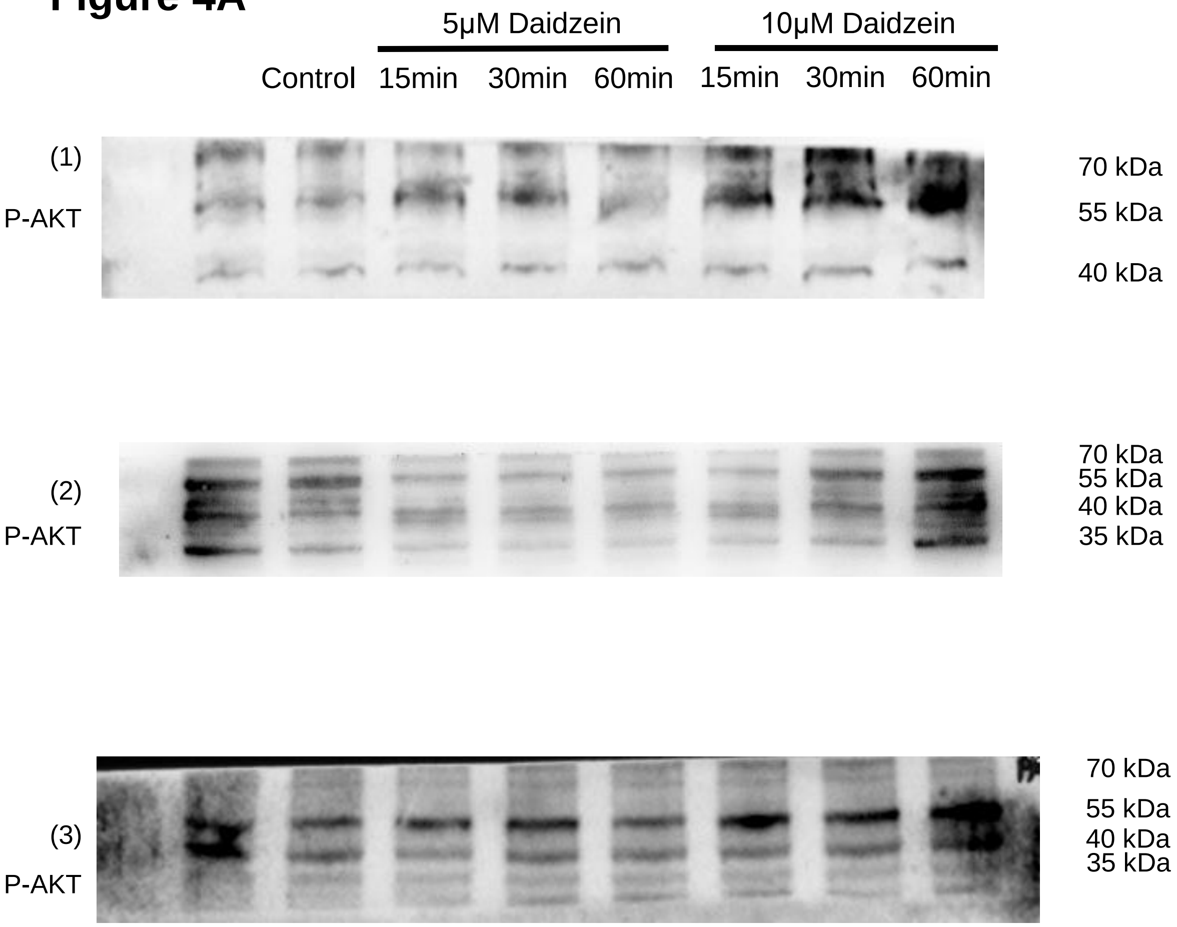
5μM Daidzein
10μM Daidzein
Control
15min
30min
60min
15min
30min
60min
(1)
P-AKT
70 kDa
55 kDa
40 kDa
(2)
P-AKT
70 kDa
55 kDa
40 kDa
35 kDa
(3)
P-AKT
70 kDa
55 kDa
40 kDa
35 kDa

Figure 4C

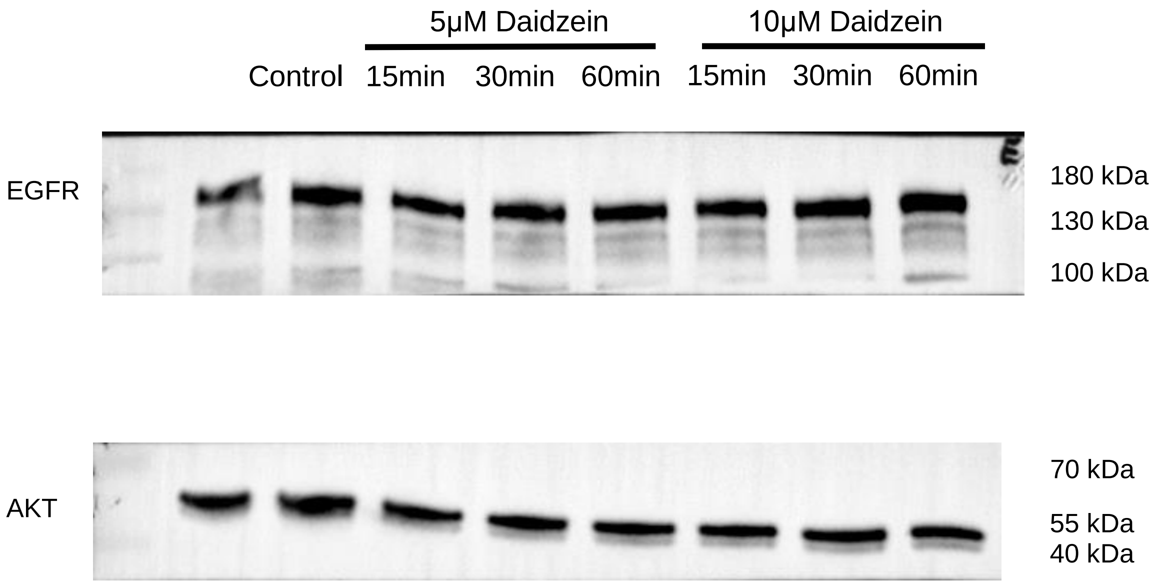
5μM Daidzein
10μM Daidzein
Control
15min
30min
60min
15min
30min
60min
EGFR
180 kDa
130 kDa
100 kDa
AKT
70 kDa
55 kDa
40 kDa

Figure 4A

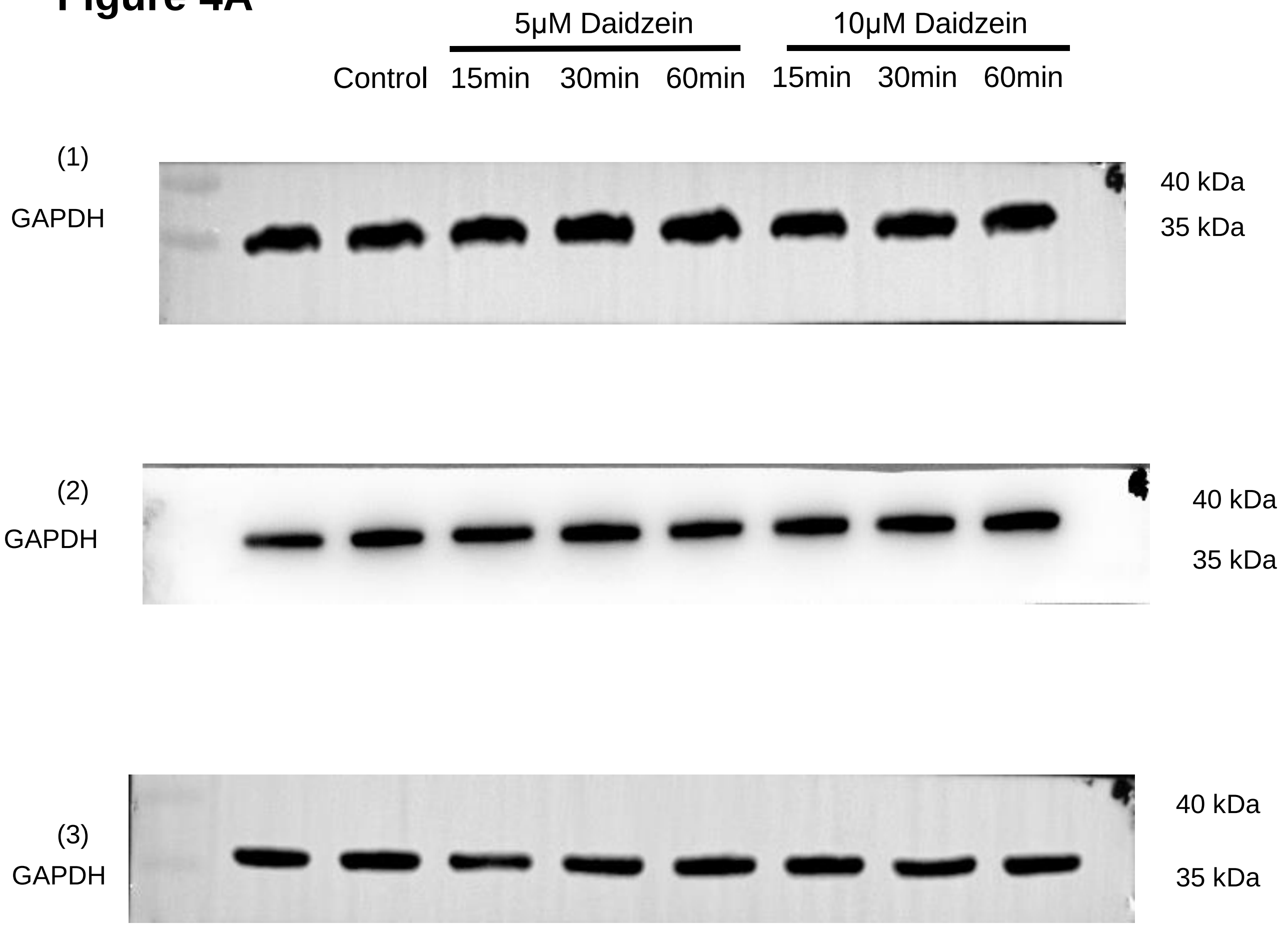
5μM Daidzein
10μM Daidzein
Control
15min
30min
60min
15min
30min
60min
(1)
GAPDH
40 kDa
35 kDa
(2)
GAPDH
40 kDa
35 kDa
(3)
GAPDH
40 kDa
35 kDa

Figure 4A

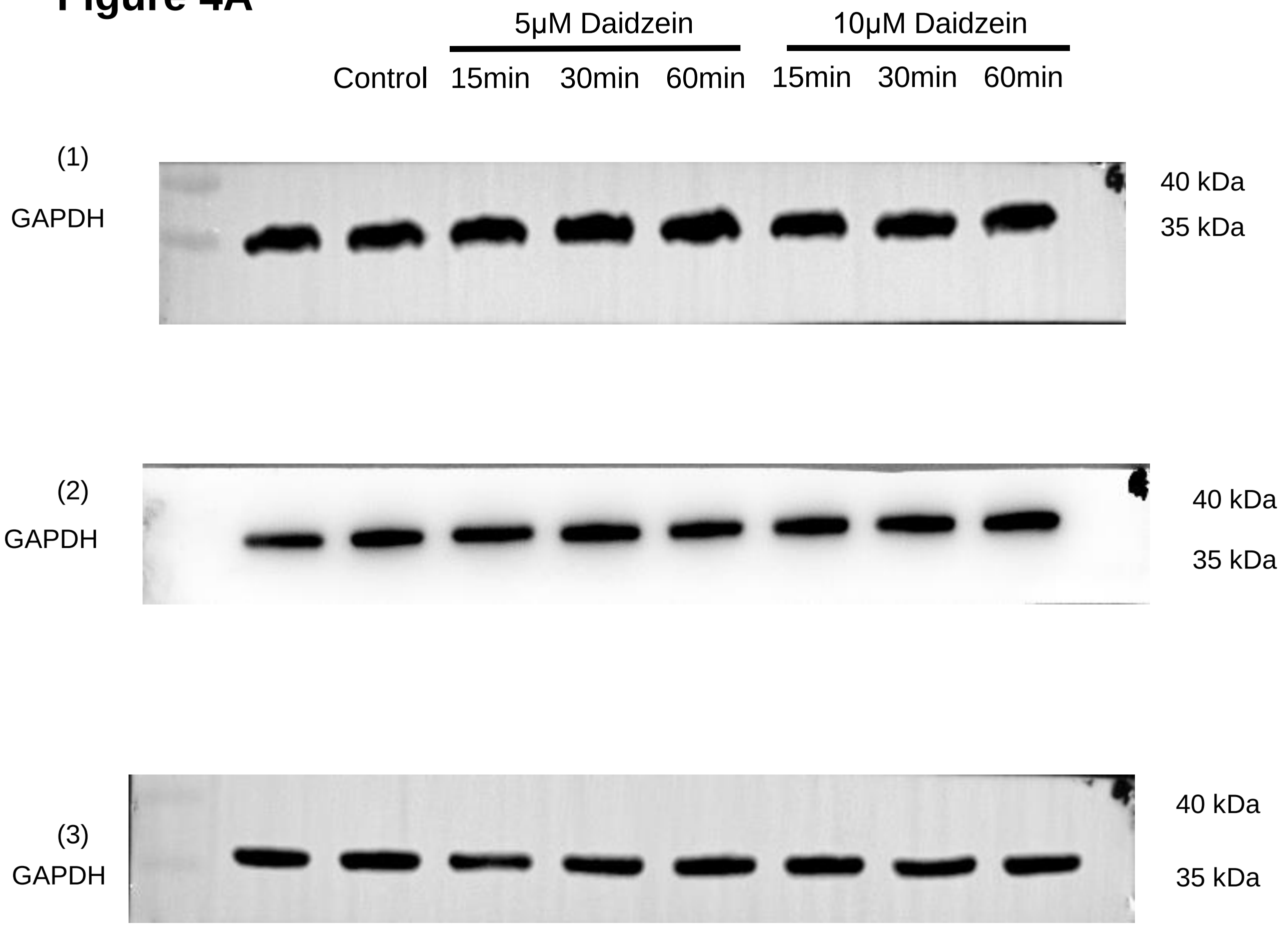
5μM Daidzein
10μM Daidzein
Control
15min
30min
60min
15min
30min
60min
(1)
GAPDH
40 kDa
35 kDa
(2)
GAPDH
40 kDa
35 kDa
(3)
GAPDH
40 kDa
35 kDa

Figure 4C

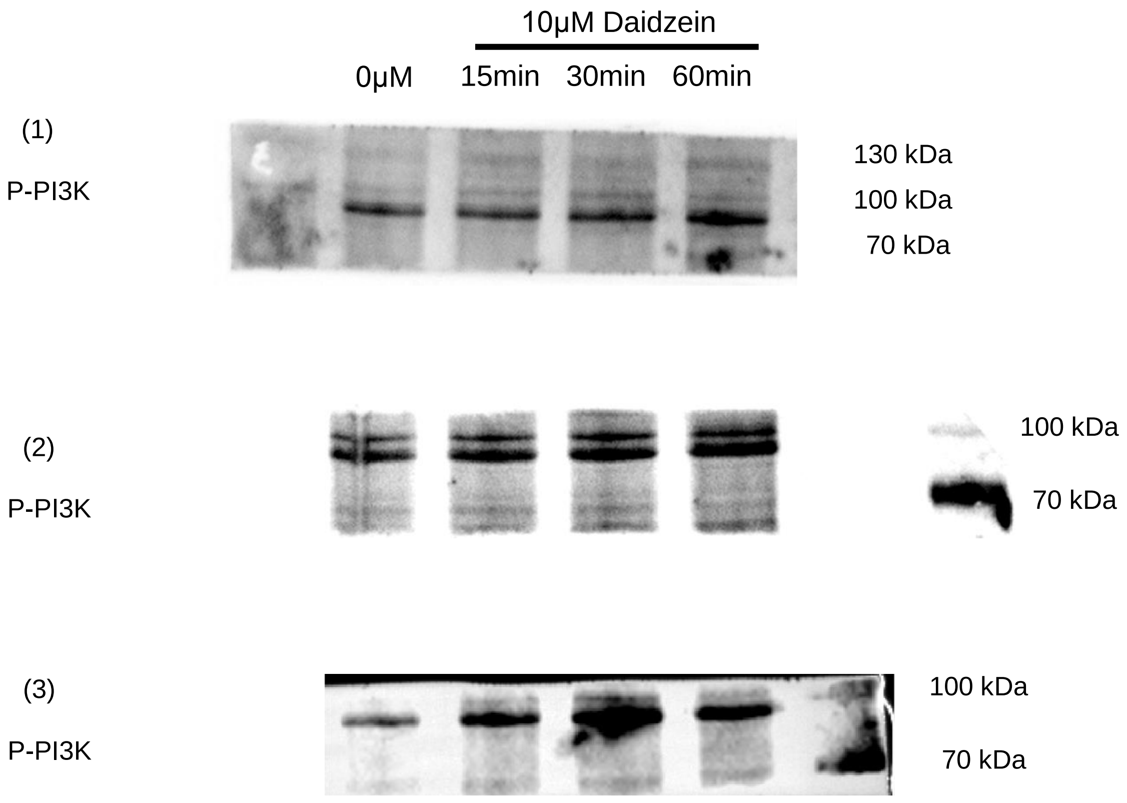
10µM Daidzein
0µM
15min
30min
60min
(1)
P-PI3K
130 kDa
100 kDa
70 kDa
(2)
P-PI3K
100 kDa
70 kDa
(3)
P-PI3K
100 kDa
70 kDa

Figure 4C

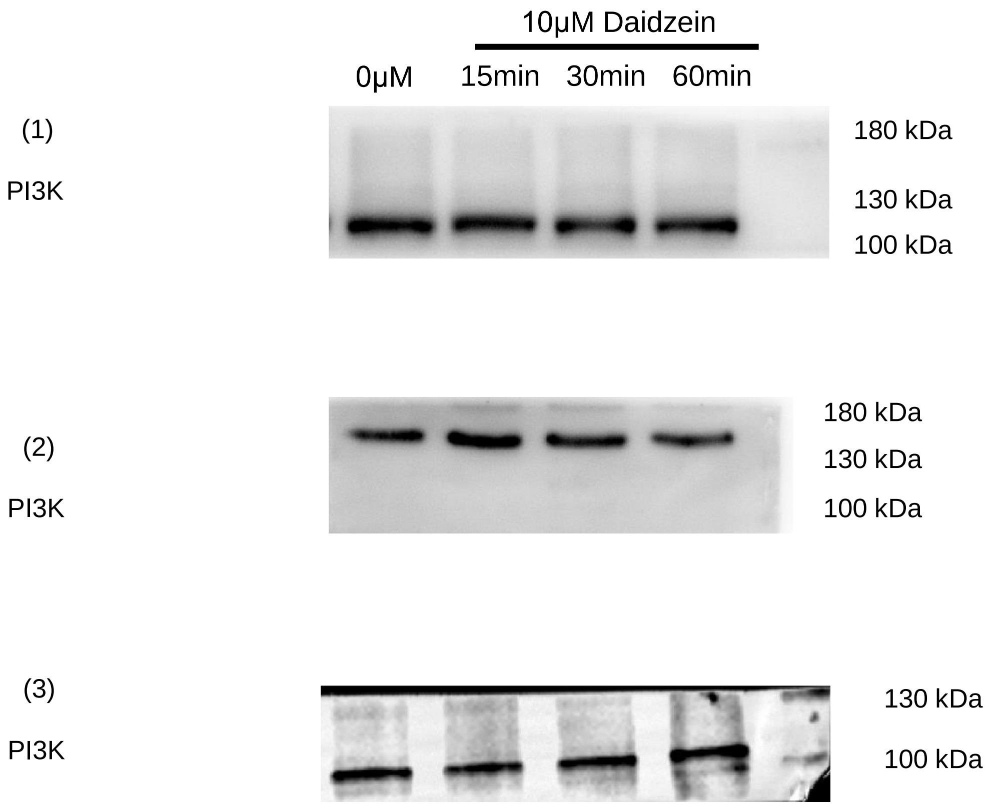
10μM Daidzein
0μM
15min
30min
60min
(1)
PI3K
180 kDa
130 kDa
100 kDa
(2)
PI3K
180 kDa
130 kDa
100 kDa
(3)
PI3K
130 kDa
100 kDa

Figure 4C

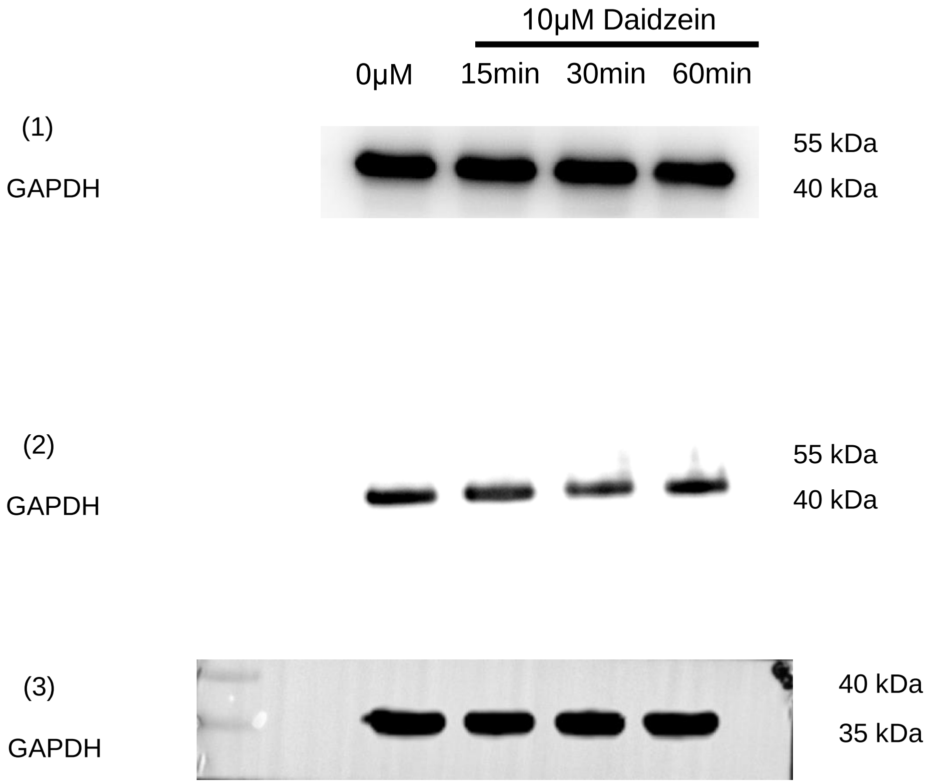
10µM Daidzein
0µM
15min
30min
60min
(1)
GAPDH
55 kDa
40 kDa
(2)
GAPDH
55 kDa
40 kDa
(3)
GAPDH
40 kDa
35 kDa

Figure 5E

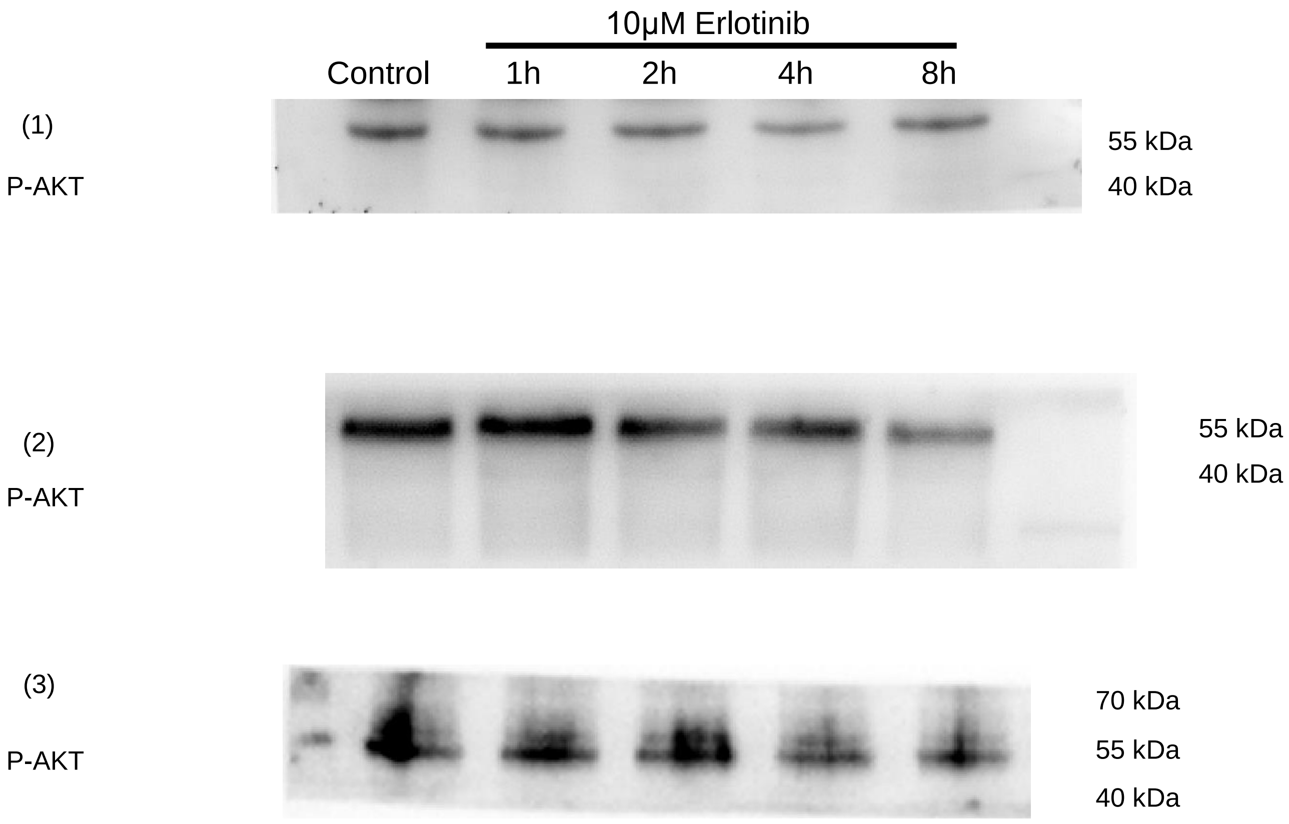
10μM Erlotinib
Control
1h
2h
4h
8h
(1)
P-AKT
55 kDa
40 kDa
(2)
P-AKT
55 kDa
40 kDa
(3)
P-AKT
70 kDa
55 kDa
40 kDa

Figure 5E

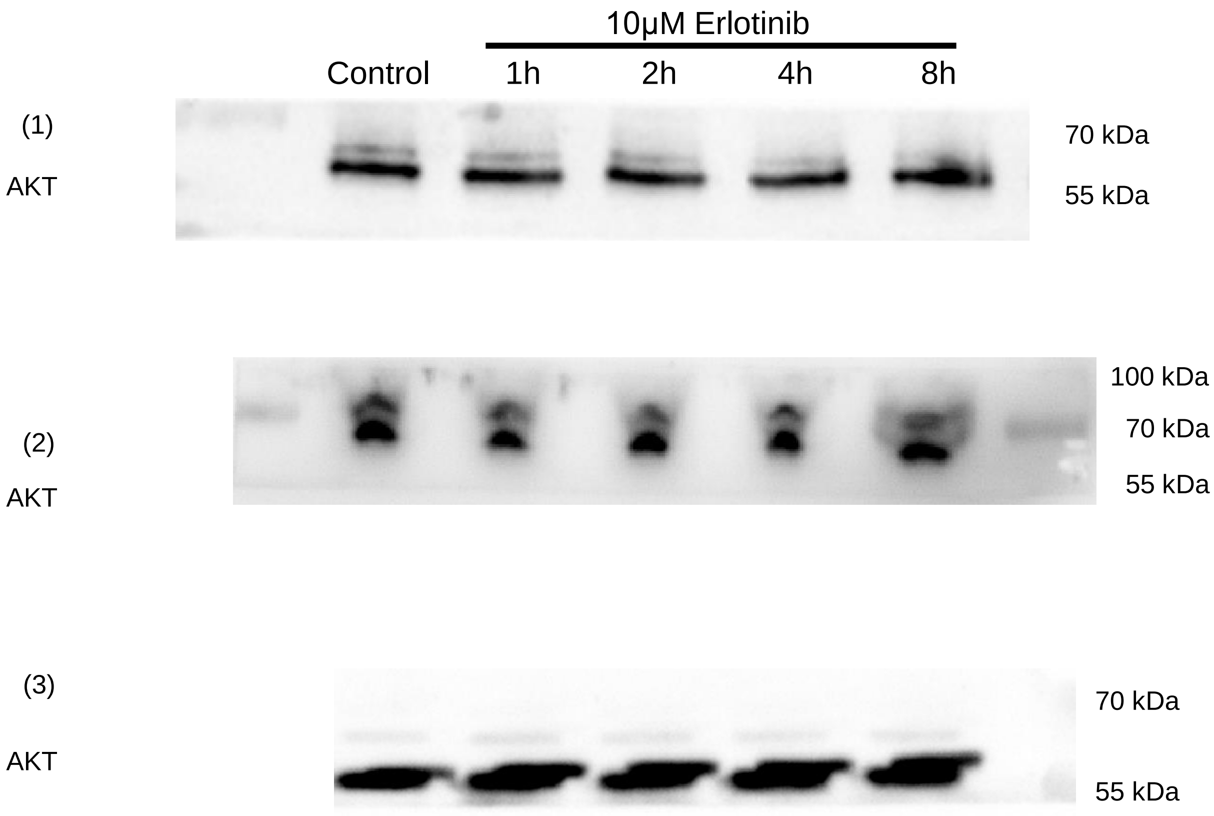
10μM Erlotinib
Control
1h
2h
4h
8h
(1)
AKT
70 kDa
55 kDa
(2)
AKT
100 kDa
70 kDa
55 kDa
(3)
AKT
70 kDa
55 kDa

Figure 5E

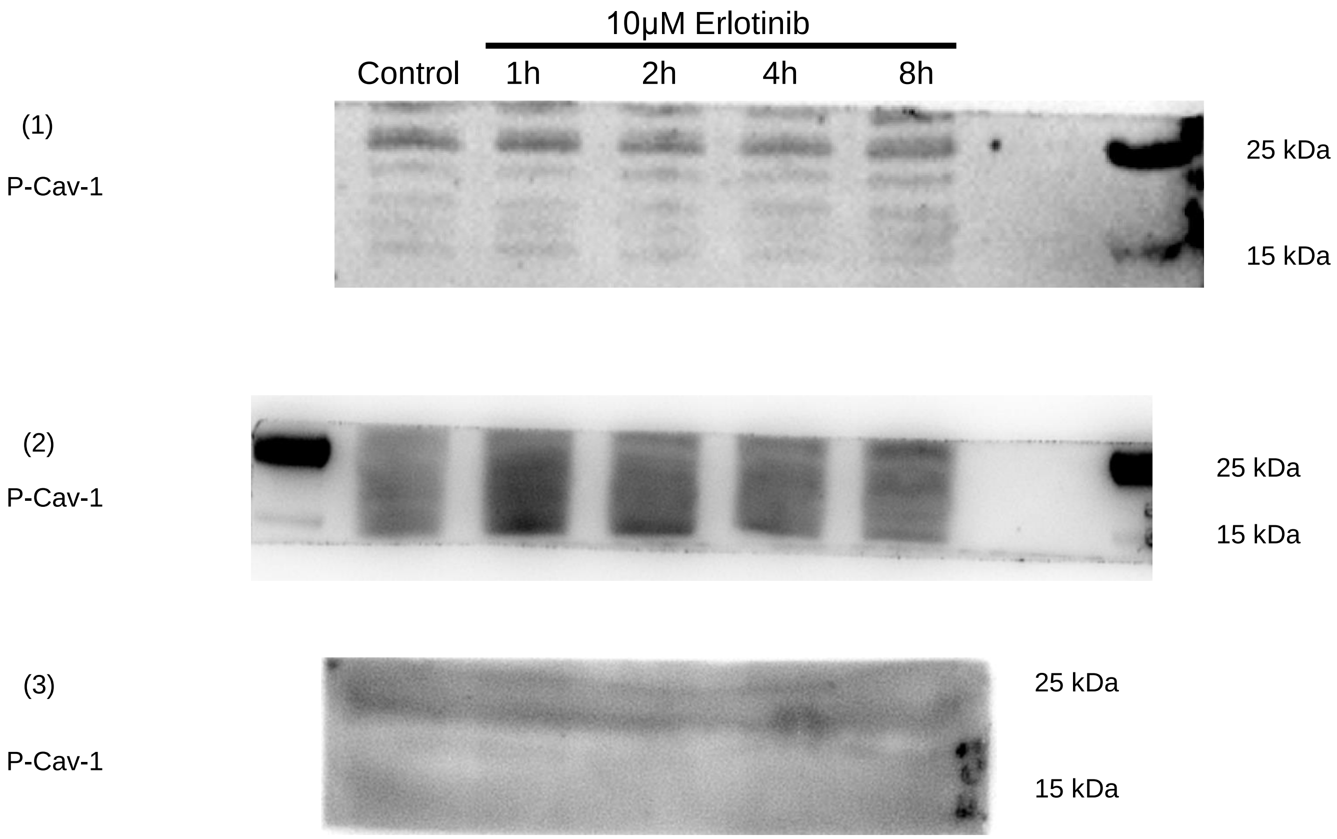
10μM Erlotinib
Control
1h
2h
4h
8h
(1)
P-Cav-1
25 kDa
15 kDa
(2)
P-Cav-1
25 kDa
15 kDa
(3)
P-Cav-1
25 kDa
15 kDa

Figure 5E

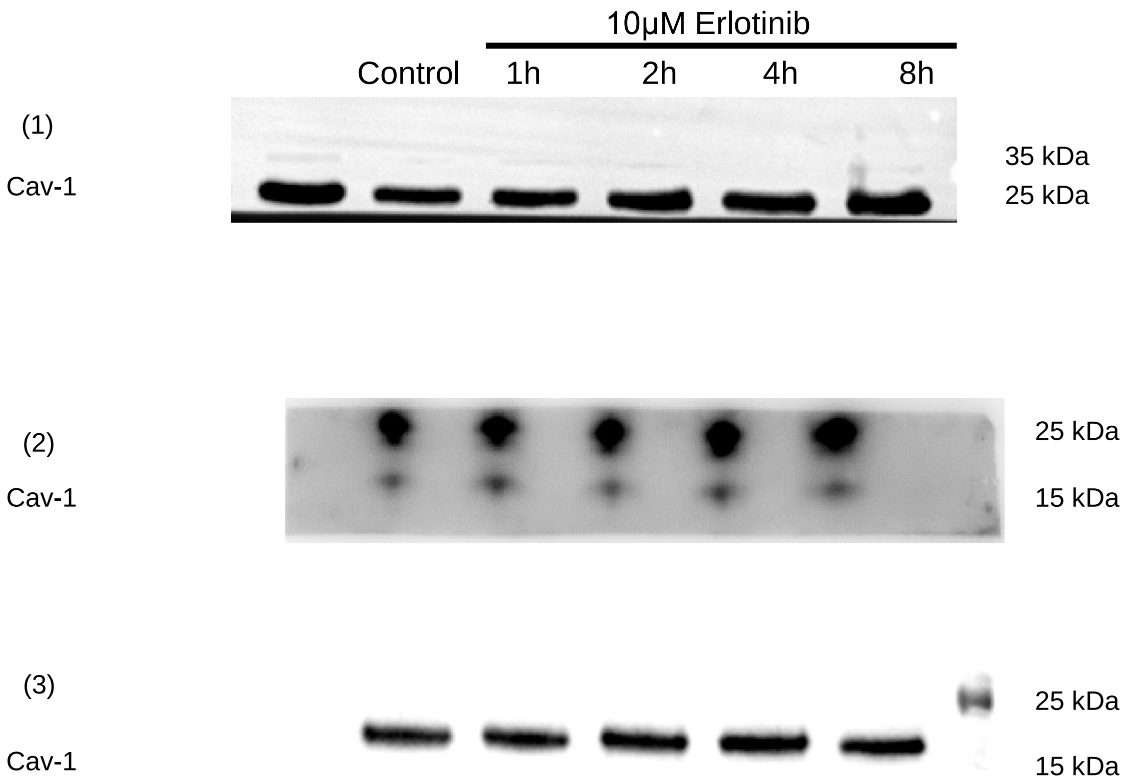

10μM Erlotinib
Control
1h
2h
4h
8h
(1)
Cav-1
35 kDa
25 kDa
(2)
Cav-1
25 kDa
15 kDa
(3)
Cav-1
25 kDa
15 kDa

Figure 5E

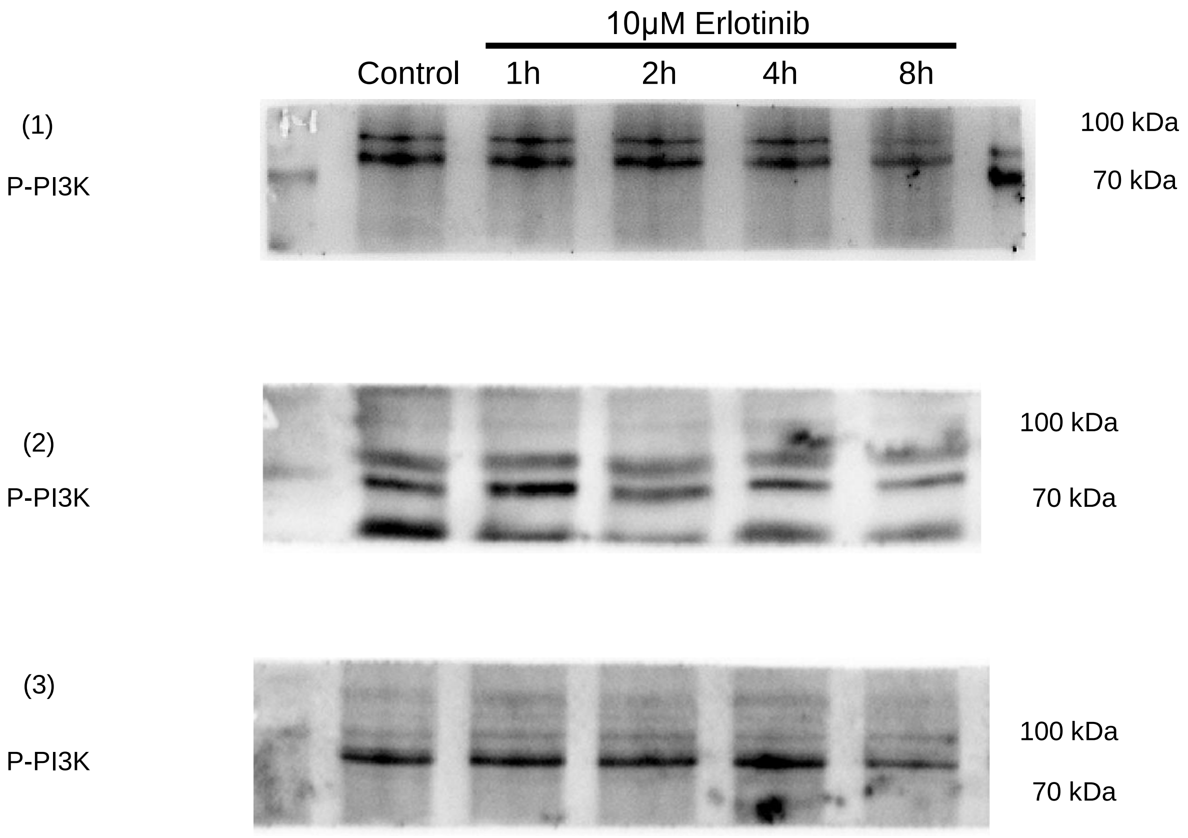

10µM Erlotinib
Control
1h
2h
4h
8h
(1)
P-PI3K
100 kDa
70 kDa
(2)
P-PI3K
100 kDa
70 kDa
(3)
P-PI3K
100 kDa
70 kDa

Figure 5E

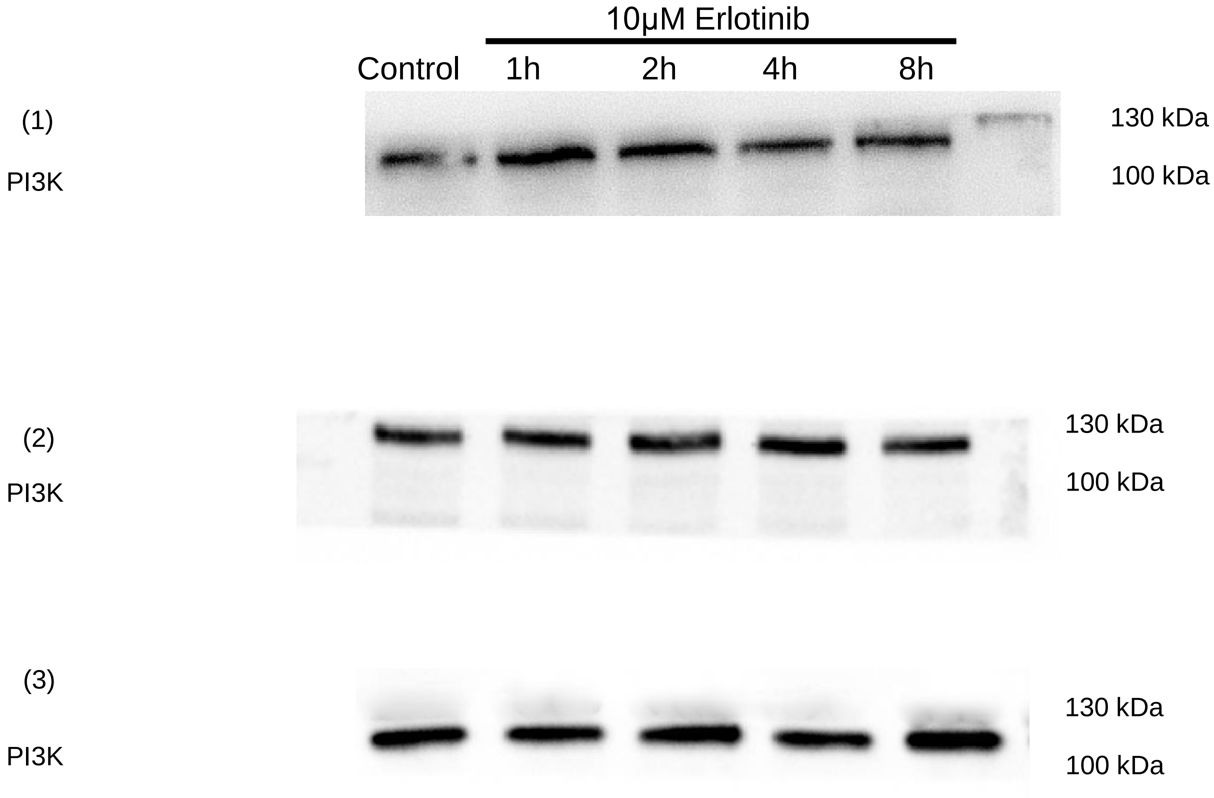
10μM Erlotinib
Control
1h
2h
4h
8h
(1)
PI3K
130 kDa
100 kDa
(2)
PI3K
130 kDa
100 kDa
(3)
PI3K
130 kDa
100 kDa

Figure 5E

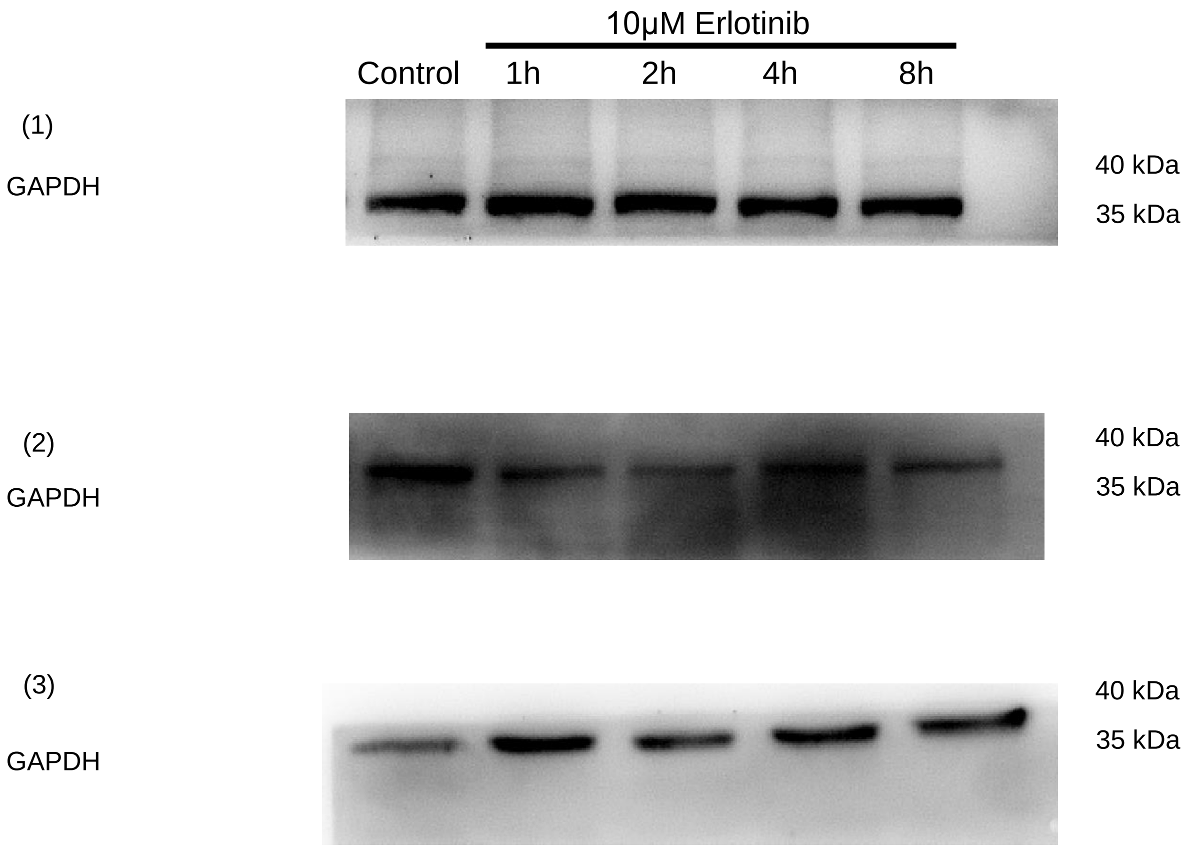
10μM Erlotinib
Control
1h
2h
4h
8h
(1)
GAPDH
40 kDa
35 kDa
(2)
GAPDH
40 kDa
35 kDa
(3)
GAPDH
40 kDa
35 kDa
